# BREADTH OF ADVERSE OUTCOMES ASSOCIATED WITH ANOREXIA/APPETITE LOSS IN OLDER POPULATIONS

**DOI:** 10.1093/geroni/igac059.1074

**Published:** 2022-12-20

**Authors:** Roger Fielding, Francesco Landi, Karen Smoyer, Thomas McRae, Lisa Tarasenko, John Groarke

**Affiliations:** Jean Mayer USDA Human Nutrition Research Center on Aging, Tufts University, Boston, Massachusetts, United States; Fondazione Policlinico Universitario Agostino Gemelli IRCSS, Rome, Lazio, Italy; Envision Pharma Group, Philadelphia, Pennsylvania, United States; Pfizer Inc, New York, New York, United States; Pfizer Inc, New York, New York, United States; Pfizer Inc, New York, New York, United States

## Abstract

The scope of the clinical problem of anorexia or appetite loss (AL) in older populations is potentially under-appreciated. A systematic literature review (SLR) was conducted following PRISMA guidelines to summarize associations of anorexia/AL in older adults with a range of clinical outcomes. Searches were run January/1/2011–July/31/2021 in PubMed, Embase®, and Cochrane databases to identify English-language studies of adults (aged ≥65 years) with anorexia/AL. Two independent reviewers screened titles/abstracts and full text against pre-defined inclusion/exclusion criteria. In all, 146 studies underwent full-text review; 58 met eligibility criteria. Most were from Europe (34/58 [58.6%]) or Asia (16/58 [27.6%]); only 3 (5.2%) were from the United States (US). Although mortality (n=18) and malnutrition (n=15) were the most reported outcomes, no US study reported on these outcomes. Other commonly reported outcomes included sarcopenia (n=7), functional status (n=6), need for increased care (n=6), and hospitalization (n=4). Significant associations of anorexia/AL were most consistently observed with mortality (17/18 studies), malnutrition (15/15 studies), and functional status (5/6 studies). Although the overall number of studies is small, associations have emerged with falls (2/3 studies), health related quality of life (2/3 studies), depression (2/2 studies), and cognition (2/2 studies). However, associations with hospitalization (1/4 studies), sarcopenia as assessed by muscle strength (2/4 studies) or by muscle mass (1/3 studies), and need for increased care (3/6 studies) were less consistent. Together, these studies highlight the breadth of adverse outcomes associated with anorexia/AL in older populations and a need for more US-based research into this common clinical problem.

